# Effect of agility ladder training with a cognitive task (dual task) on physical and cognitive functions: a randomized study

**DOI:** 10.3389/fpubh.2023.1159343

**Published:** 2023-06-21

**Authors:** Vivian Castillo de Lima, Luz Albany Arcila Castaño, Ricardo Aurélio Carvalho Sampaio, Priscila Yukari Sewo Sampaio, Camila Vieira Ligo Teixeira, Marco Carlos Uchida

**Affiliations:** ^1^Laboratory of Applied Kinesiology, Faculty of Physical Education, State University of Campinas, Campinas, Brazil; ^2^Department of Physical Education, Federal University of Sergipe, São Cristóvão, Brazil; ^3^Department of Occupational Therapy, Federal University of Sergipe, São Cristóvão, Brazil; ^4^NIH Biomedical Research Center, University of Maryland, Baltimore, MD, United States

**Keywords:** aging, cognitive function, executive function, dual-task, physical function

## Abstract

**Introduction:**

Agility training (AT) is used to improve neuromuscular performance and dynamic balance, which are crucial for the physical function of older adults. Activities of daily living, which decrease with age, involve tasks that simultaneously require motor, and cognitive abilities and can be considered dual tasks.

**Methods:**

This study investigates a training program's physical and cognitive effects using an agility ladder on healthy older adults. This program consisted of 30-min sessions twice per week and lasted for 14 weeks. The physical training included four different sequences with progressive difficulty levels, while the cognitive training (CT) included different verbal fluency (VF) tasks for each physical task. Sixteen participants (mean age of 66.9 ± 5.0 years) were allocated to two groups: AT alone (AT) and dual-task training (AT combined with CT [AT + CT]). Assessments were performed before and after 14 weeks of interventions using physical functional tests (e.g., Illinois agility test, five times sit-to-stand test, timed up and go [TUG], and one-leg stand) and cognitive tests (cognitive TUG, verbal fluency, attention, and scenery picture memory test).

**Results:**

After this period, both groups had significant differences in physical performance, muscle power, agility, static and dynamic balance, and short-term memory, whereas only the AT + CT group improved phonological verbal fluency, executive function (TUG combined with a cognitive task), attention (trail-making test-B), and short-term memory (scenery picture memory test).

**Conclusion:**

Indicating that only the group that received direct cognitive training had better enhanced cognitive function.

**Clinical trial registration:**

www.ClinicalTrials.gov, identifier: RBR-7t7gnjk.

## Introduction

Aging is associated with neuromuscular, cardiovascular, and central nervous system decline (1) and has been shown to impair physical function and decrease muscle mass, strength, and power (2), leading to functional limitations associated with independence and autonomy reduction, decreasing, consequently, the quality of life in older adults. In this respect, the World Health Organization (WHO) reported that the age-related decrease in neuromuscular and cognitive function limits the execution of multiple tasks, such as walking and talking on the cell phone, or walking and watching traffic lights (3). The performance of multiple tasks may demand a special effort to coordinate and modulate the physical (physical action, e.g., walking) and cognitive task (cognitive action, e.g., attention and executive function), simultaneously. The capacity to modulate these functions is reduced with age. Moreover, functional decline and geriatric syndromes associated with non-communicable diseases reduce the capacity to perform activities of daily living (1, 4).

The modulation of dual tasks or multiple tasks requires divided attention in different actions, physical or cognitive tasks. This modulation may have interfered with gait and postural control, which may cause the risk of falling (3). Therefore, new strategies, and therapies have been developed as aerobic training, stepping training (agility), and resistance training in dual task (physical task + cognitive task, simultaneously), preserving neuromuscular and cognitive function (5–9).

Agility training (AT) is the other physical function that can increase neuromuscular performance and dynamic balance (10). Agility has classically been defined as the simple ability to change direction rapidly. According to Young et al., agility involves a rapid displacing of the center of mass by changing direction or speed when reacting to a stimulus (11). On the other hand, this kind of physical activity was used as a methodology to improve physical function in older adults (12).

Yamada et al. (9) trained older adults during 60 min of rhythmic step exercise (step to multidirectional) with a cognitive task (reaction time and short memory), simultaneously for 24 weeks. This study showed that the program promotes training across different modalities (motor and cognitive functions), leading to improvements in tasks that need more attention, such as walking while performing the cognitive task (i.e., counting numbers aloud in inverse order) (9).

The improvement of divided attention (cognitive and/or physical task) benefits multiple functions, depending on the trained capacities. The physical training program provides physical adaptation (e.g., resistance training improves strength and power muscle), and the cognitive training (CT) program provides cognitive adaptations (e.g., verbal fluency [VF] training improves VF ability) simultaneously (6, 9, 13). Castaño et al. (6) performed resistance exercises in two conditions, with and without a cognitive task. The dual task (resistance exercise + cognitive task, simultaneously) used VF as a cognitive task. After 16 weeks of intervention, the study showed improvements in executive function and physical capacities.

We proposed that agility ladder training with VF (cognitive task) might help improve dual-task abilities (physical and cognitive function, simultaneously). For this reason, the effect of a 14-week exercise program involving AT with and without cognitive tasks on physical and cognitive functions in community-dwelling older adults was evaluated. We hypothesized that physical function would increase in both groups and cognitive function would improve only in the dual-task group.

## Materials and methods

### Study design

This two-arm, parallel, randomized, controlled trial compared the effects of AT and AT + CT (two groups) on cognitive function and physical performance in community-dwelling older adults without cognitive decline. Participants were recruited through advertisements in public sports areas located in Campinas, state of São Paulo, Brazil. After checking for eligibility, the participants were randomly allocated into the intervention groups using computer-generated random numbers (https://www.randomizer.org). The numbers were generated using “Math. Random” with a complex algorithm that gives the appearance of randomness. The education and literacy of the participants were evaluated through demography questionnaires.

The study was approved by the Research Ethics Committee of the University of Campinas (UNICAMP) (Protocol No. 2479761) and followed the ethical guidelines of the Declaration of Helsinki and Resolution 466/12 of the Brazilian Health Council. Participants were informed about the study procedures and objectives before giving written informed consent.

### Eligibility criteria

We included participants aged >60 years old who were cognitively healthy, physically independent, and able to perform the physical function tests.

We excluded subjects who had started a structured physical activity program 2 months before the beginning of the study or participated in other exercise programs during the study period, and individuals with a clinical diagnosis of cardiovascular (e.g., acute myocardial infarction and transient ischemic disease), pulmonary (e.g., emphysema), neurological, psychiatric (e.g., Parkinson's, dementia or Alzheimer's disease), skeletal muscle disorders, and cognitive disorders (MMSE score was used as criteria of exclusion <24). The participants who were absent for more than 10% of the exercise sessions were also excluded from the study analysis.

The Mini-Mental State Examination (MMSE) was used as cognitive screening according to the Brazilian education level (14). The MMSE assesses spatial orientation, short memory, attention, and calculation, as well as the ability to name objects, follow commands, write a sentence, and reproduce a complex drawing (15).

All experimental procedures were conducted at the School of Physical Education of UNICAMP from February 2018 to June 2018.

## Interventions

**Figure 1:**
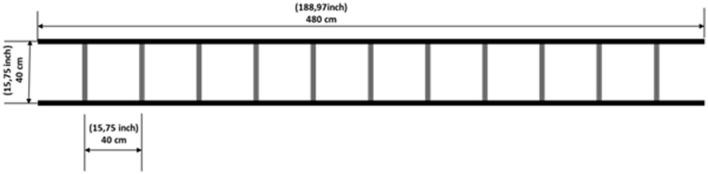
Agility ladder with a length of 4.8 m and 12 rungs (40 cm × 40 cm).

AT and AT + CT were performed twice a week for 14 weeks in two phases: a 2-week familiarization period followed by a 14-week training period. Exercise sessions lasted approximately 30 min, including a 10-min warm-up, a 15-min exercise session (main part), and a 5-min cooldown. All sessions were conducted in groups of two or three people, with a distance of 2 m between the participants to avoid training interference. The participants of the AT + CT group were assigned the same cognitive task but in a different order. An agility ladder adapted from sports training was used in exercise sessions (Figure 1). Participants were instructed to step in the squares and not on the rungs and performed all activities under the supervision of an experienced trainer, who had 5 years of experience and supervised all the training sessions.

The familiarization period was designed to adapt participants to the laboratory setting, exercise sequences, and perception of effort (rating of perceived exertion). Participants were instructed to perform each exercise sequence round trip on the agility ladder for 30 s.

**Figure 2:**
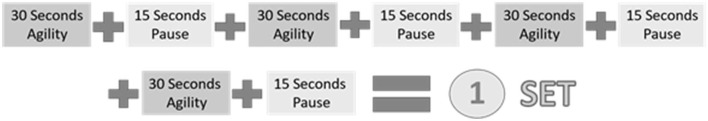
Exercise sets. Each exercise session (e.g., sequence A) included four subsets, each with a 30-s exercise period and a 15-s rest.

The duration of each session was equal for both groups (AT and AT + CT). Participants completed the same exercise sequence during the familiarization period. At week 6, the exercise difficulty was increased by substituting sequences 2A and 3A for 2B and 3B, respectively, and at week 10, difficulty increased by adding 30 s to each sequence (Figure 2). The sequences were considered with different combinations of the foot stepping on the ladder, as previously published by our group members (16).

Participants performed four sequences 1, 2 (2A or 2B), 3 (3A or 3B), and 4 for 30 s each, followed by 15 s of rest (Table 1). The physical exercise (agility ladder) sequence was performed in the same order by both groups (AT and AT + CT). Each session lasted 12 min from weeks 1 to 10 (3 sets to each sequence) and 15 min at weeks 11 and 12 (4 sets to each sequence), and more details were previously published (16).

**Table 1:** Training sessions outlook.

**Description**	**Quantity**															
**Sequences**	**1**				**2A or 2B**				**3A or 3B**				**4**			
Sets	1	2	3	4	1	2	3	4	1	2	3	4	1	2	3	4
Duration set (s)	30	30	30	30	30	30	30	30	30	30	30	30	30	30	30	30
Rest sets (s)	15	15	15	-	15	15	15	-	15	15	15	-	15	15	15	-
Rest sequences (s)	60				60				60				60			

Sequence (combination of the footsteps on the ladder) = 1, 2 (2A or 2B), 3 (3A or 3B).

The AT + CT group performed agility training concurrently with a cognitive task (e.g., VF). The participants were instructed to say aloud as many words of a specific category as possible in each subset. The difficulty level of the cognitive task was increased monthly by changing word categories, from general to specific, while phonemic (e.g., words beginning with vowels letters and consonants) or semantic (e.g., people names, sports, clothes, male names, aquatic sports, and winter clothes) categories were changed in each subset (Table 2). Participants were encouraged to not repeat words in each subset.

**Table 2:** Examples of semantic (e.g., 1st set—color and 2nd set—country) and phonological (e.g., 3rd set—M letter and 4th set—R letter) categories used for the verbal fluency test.

**Sequence (A)**	**Time**									
	**30 Seconds**									
1^st^ Set	Green	Blue	Yellow	Red	Orange	Purple	White	Black	Gray	Pink
2^nd^ Set	Brazil	Japan	Argentina	Italy	Colombia	China	Mexico	France	Paraguay	Spain
3^rd^ Set	Mouse	Mini	Max	Math	Make	Map	Made	Medium	Memory	Meet
4^th^ Set	Rule	Run	River	Route	Ring	Ready	Read	Rat	Rain	Right

### Measures

Assessments were performed before (at the baseline) and after the intervention (14 weeks), and each evaluation lasted for 2 days. Physical performance was assessed on the 1^st^ day, and cognitive function was evaluated on the 2^nd^ day.

### Physical function

The following physical function tests were performed: (1) Walking speed (WS) at a normal and fast pace, (2) five times sit-to-stand test (5XSTS), (3) timed up and go (TUG), (4) isometric handgrip strength (IHG), (5) one-leg stand (OLS), and (6) Illinois agility test (IAT).

### Walking speed

The WS test required walking 12 m at a normal and fast pace. Before the test, both feet remained on the starting line. The stopwatch was started when one foot reached the 1-m line and was stopped when one foot reached the 11-m line. The first and last 1-m stretches were used for acceleration and deceleration, respectively, and therefore were not considered (17). The fastest time of two trials (in m/s) was used for the present analyses.

### Five times sit-to-stand test

This test comprises rising from and seating on an armless chair (total height, 87 cm; seat height, 45 cm; seat width, 33 cm) five times as fast as possible with arms crossed in front of the body. A stopwatch (1/100 second accuracy) was started when the participant raised the hip from the chair and stopped when the participant sat down for the fifth time (18).

### Timed up and go

Upon hearing the command “go,” the participants were required to get up from a chair without using their arms, walk as fast as possible along a 3-m straight line demarcated on the floor, turn around, return to the original position, and sit down on the chair again (19).

### Isometric handgrip strength

The IHG was measured using a Jamar^®^ dynamometer with participants sitting on a chair with shoulders adducted, elbows flexed at 90° beside the trunk, and wrists in a neutral position. The contralateral arm remained relaxed beside the trunk. The study subjects were asked to squeeze the handgrip as hard as they could for 4 to 6 s using the dominant hand. The highest test–retest reliability for each test was achieved, and 1 min to rest between retests was provided. The mean of three trials was used (20). Relative IHG was calculated by dividing IHG by the BMI.

### One-leg stand

This test was performed with participants standing on one foot, the contralateral knee flexed at 90°, arms folded across the chest, and head straight. The stopwatch was started when one foot was raised off the floor and stopped when the foot touched the floor again. The test was performed in both legs, and the highest score was used in the analysis (21).

### Illinois agility test

**Figure 3:**
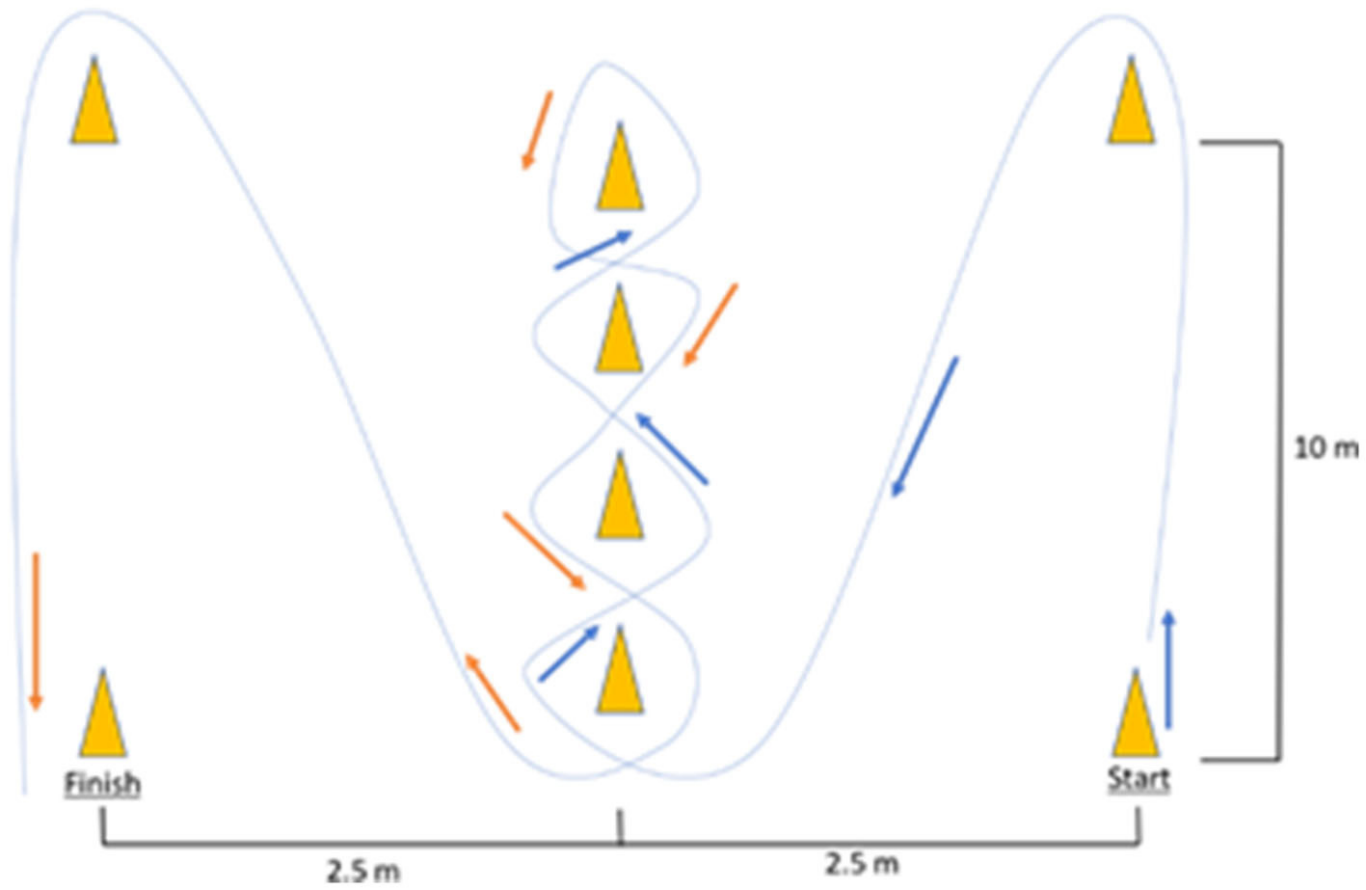
Illinois agility test.

The participant was asked to walk as fast as possible (i.e., move quickly) through obstacles in multiple directions but not run. The course was marked in the corners by four cones (start, finish, and two turning cones) and four central cones spaced 3.3 m apart. The participants were instructed to walk in a straight line from the start line to the first turning cone located 10 m apart and from this site to the first central cone, weave back and forth through the four central cones, and walk from the first central cone to the second turning cone located on the far right and from this point to the finish line (22) (Figure 3).

### Cognitive function

Cognitive function was assessed using VF (23), dual task, TUG combined with a cognitive task (TUG-cog) (24), trail-making test (TMT) (25), and the scenery picture memory test (SPMT) (26). Below we describe the tests mentioned above:

### Verbal fluency test

VF was assessed using phonological and semantic tests. Participants were requested to name as many animals (semantic domain) and words that began with the letter “A” (phonological domain) as possible for 2 min (1 min each). The scores of the VF domains (phonological [VFP] and semantic [VFS]) were calculated as the sum of all the words that were evoked for 1 min (23).

### TUG-cog

TUG-cog is a test that evaluates the divided attention, physical function, and cognitive function, simultaneously (walking [TUG test] + VF [cognitive task]). In this study, participants were required to say the names of animals out loud during the execution of TUG (27). Time started when participants got up from the chair and stopped when the participant returned to the chair and sat down. The result is shown in the second part.

### Trail-making test

The TMT provides information on visual search, scanning, speed of processing, mental flexibility, and executive functions. The TMT was divided into two parts: TMT-A and TMT-B. TMT-A consisted of drawing a line connecting a sequential set of numbers (1–25), whereas TMT-B consisted of drawing a line connecting sequential numbers (1–13) and letters (A–L) and alternating between numbers and letters (e.g., 1a, 2b, 3c, and 4d). The test should be performed as quickly as possible (28). The final score is the total time spent finishing the connection between letters and numbers.

### Scenery picture memory test

This test is based on the memorization of an image, requiring attention and short-term memory. The image of a living room containing 23 objects was drawn on a piece of paper. The participants were instructed to examine the image for 1 min and mention which elements they remembered. The total score corresponded to the number of items recalled (26).

### Statistical analysis

Descriptive data were shown as mean ± standard deviation (SD). Continuous variables (age, formal education years, height, BMI, and MMSE scores) were compared using the Mann–Whitney *U*-test. Gender was compared using the chi-square test.

The effect of exercise on the study groups (AT and AT + CT) was analyzed using a two-way analysis of variance (ANOVA). Tukey's *post hoc* tests were used to assess which group or time showed significant differences. Effect size (ES) was calculated using each variable of the post-training score (value) minus the pre-intervention (baseline), and then divided by the pre-intervention (baseline). An ES of 0.00–0.19, 0.20–0.49, 0.50–0.79, and ≥0.80 were considered marginal, small, moderate, and large, respectively (29). Delta was also calculated [(baseline value – post-training value)/baseline value × 100]. ES and delta were calculated for all the variables, and *post hoc* was performed only for variables that presented a difference. The relationship between AT and cognitive test scores after training was assessed using Pearson's correlation. The data were analyzed using Statistical Analysis System version 9.4 for Windows (SAS Institute Inc., Cary, NC, USA). Statistical significance was set at a *p*-value of < 0.05.

## Results

**Figure 4:**
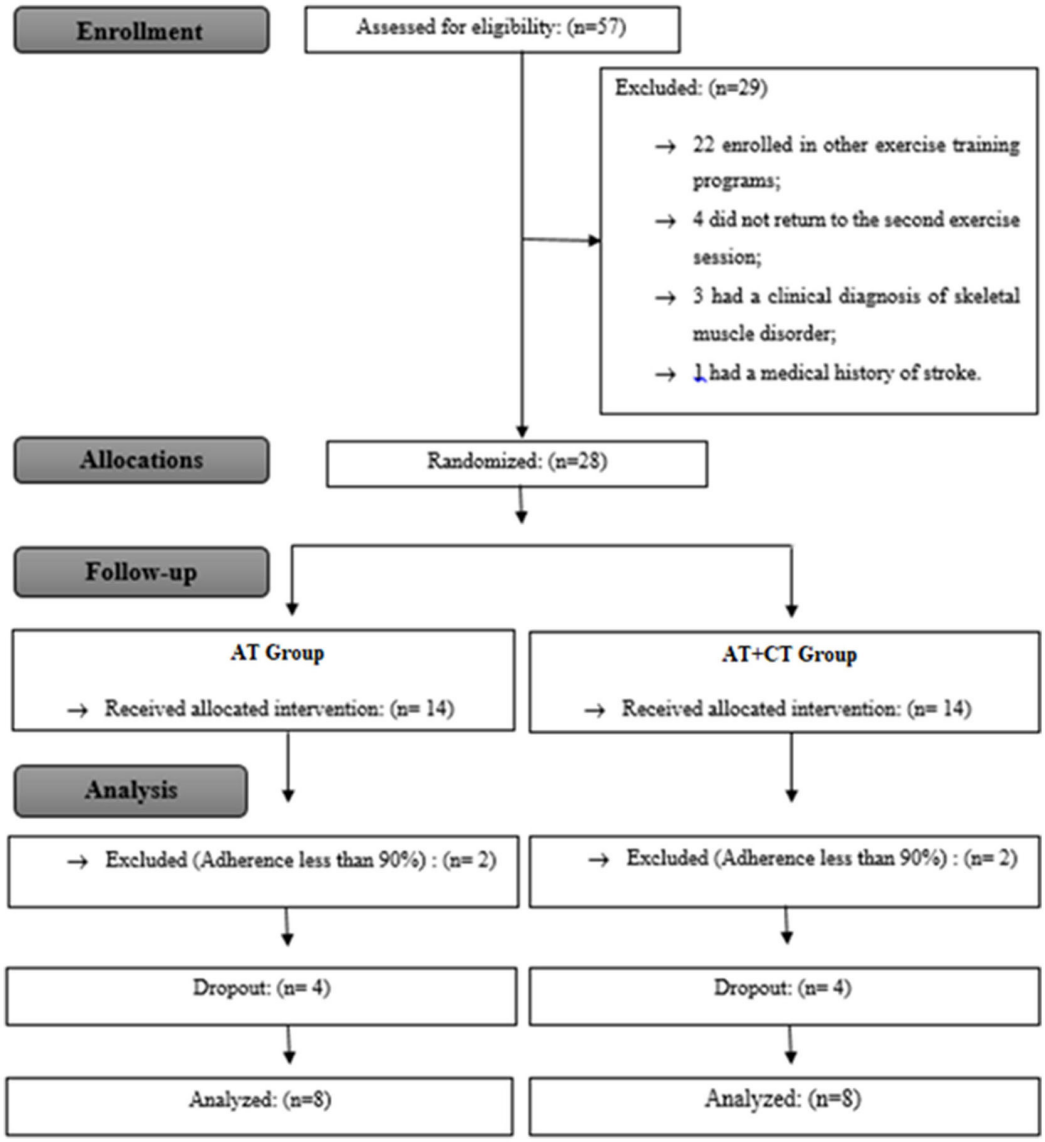
Study flowchart: AT, agility training; AT + CT, AT combined with cognitive training.

A total of 57 older adults were recruited. In total, 27 participants were excluded according to the eligibility criteria, and 28 were randomly allocated into the AT and AT + CT groups. Twelve participants were excluded from the analysis: four had <90% adherence to the training sessions, and we had eight dropouts. Hence, 16 subjects were included in the study and completed the training program (Figure 4).

There were no significant differences between groups in the baseline characteristics, as shown in Table 3.

**Table 3:** Baseline comparison of both groups characteristics.

**Variables**	**AT (n=8)**	**AT+CT (n=8)**	***p*-value**
Age (years)	66.6 ± 5.7	67.1 ± 4.6	0.64
Formal education (years)	12.6 ± 2.0	11.4 ± 4.8	0.96
Male gender (*n*, %)	3 (37.5%)	2 (25.0%)	0.72
Height (cm)	1.6 ± 0.1	1.6 ± 0.1	0.57
Body mass (kg)	70.6 ± 12,7	70.7 ± 7.2	0.87
BMI (kg/m^2^)	26.8 ± 4.3	26.7 ± 3.0	0.67
MMSE (points)	28.3 ± 1.7	27.2 ± 3.3	0.57

AT, Agility training; BMI, body mass index; CT, Cognitive training; MMSE, Mini-Mental State Examination.

AT + CT improved the scores of TUG-cog, phonological VF, and SPMT, whereas AT had better scores in SPMT after the training. Only for TUG-cog, ANOVA showed time x group interaction (*p* = 0.019) between AT + CT and AT. For the dual-task training, the cognitive functions improved by approximately 39, 32, and 7% in the tests TUG-cog, phonological VF, and SPMT (short-term memory), respectively. AT enhanced short-term memory, which is indicated by an increase of approximately 20% in SPMT (Table 4).

**Table 4:** Effects of AT and AT+CT on cognitive functions and comparison between groups.

**Cognitive domains**	**AT**+**CT (*****n*** = **8)**				**AT (*****n** =* **8)**				**Time × Group**	**Time**	**Group**
	**Pre**	**Post**	**ES**	Δ**%**	**Pre**	**Post**	**ES**	Δ**%**			
TUG-cog (s)	9.5 ± 3.0	5.8 ± 0.5^*^^#^	1.4	38.9	7.5 ± 1.1	6.5 ± 1.0	1.0	13.3	0.0196	0.0004	0.2867
TMT-A (s)	47.3 ± 6.1	41.3 ± 13.2	0.5	2.65	37.7 ± 11.6	38.7 ± 10.1	0.1	9.6	0.4765	0.6531	0.1677
TMT-B (s)	130.7 ± 51.3	111.3± 63.2	0.5	18.4	90.4 ± 30.6	84.2 ± 24.8	0.2	6.8	0.1936	0.0937	0.2854
VFP (words/min)	12.4 ± 4.6	16.4 ± 6.6^*^	0.8	32.2	13.4 ± 2.9	13.8 ± 4.4	0.1	2.98	0.142	0.0514	0.7056
VFS (words/min)	19.7 ± 8.2	19.6 ± 8.4	0.1	0.50	18.9 ± 3.5	19.6 ± 6.1	0.2	3.7	0.8699	0.9022	0.8699
SPMT (points)	16.3 ± 2.7	17.5 ± 3.3^*^	0.4	7.3	14.9 ± 3.4	17.9 ± 3.2^*^	1.0	20.1	0.1738	0.0037	0.7354

* Main effect of time (*Post-hoc*), *p* ≤ 0.05.

# Main effect of group. AT, Agility training; CT, Cognitive training; ES, effect size; SPMT, Scenery Picture Memory Test; TMT-A, Trail Making Test A; TMT-B, Trail Making Test B; SVF, semantic verbal fluency; PVF, phonological verbal fluency.

The effects of AT + CT and AT on physical performance are shown in Table 5. The time effect (pre-post) was observed for both intervention groups (*p* < 0.05). AT + CT and AT increased the scores of WS at a normal pace, 5XSTS, TUG, IHG, relative IHG, and IAT. The AT + CT and AT groups improved physical function, with an increase in walking speed at a normal pace [WS Δ(%) 15, 15], power muscle [5XSTS Δ(%) 27, 29], dynamic balance [TUG Δ(%) 27, 22], isometric strength muscle [absolute IHG Δ(%) 21, 20], and agility function [IAT Δ(%) 13, 11], respectively (Table 5).

**Table 5:** Effects of AT and AT+CT on physical function.

**Variables**	**AT (*****n** =* **8)**				**Time** × **Group**				**Time**	**Group**	
	**Pre**	**Post**	**ES**	Δ **(%)**	**Pre**	**Post**	**ES**	Δ **(%)**			
Usual WS (m/s)	1.3 ± 0.1	1.5 ± 0.1^*^	1.4	15.3	1.3 ± 0.1	1.5 ± 0.2^*^	1.6	15.3	0.9999	0.0002	0.9999
Fast WS (m/s)	1.8 ± 0.2	1.9 ± 0.2^*^	0.9	5.5	1.9 ± 0.2	1.9 ± 0.2	0.4	0.0	0.1686	0.0009	0.4942
5XSTS (s)	11.1 ± 2.2	7.9 ± 1.2^*^	2.0	27.4	10.2 ± 1.9	7.4 ± 1.0^*^	2.1	28.8	0.6871	0.0001	0.3428
TUG (s)	8.0 ± 1.2	5.8 ± 0.8^*^	2.4	27.5	7.4 ± 1.2	5.8 ± 0.7^*^	1.8	21.6	0.4039	0.0001	0.5287
Absolute IHG (kg)	23.9 ± 5.1	28.8 ± 6.3^*^	0.9	20.5	24.6 ± 9.3	29.5 ± 10^*^	0.5	19.9	0.9999	0.0002	0.8049
Relative IHG (kg/BMI)	0.9 ± 0.3	1.1 ± 0.3^*^	0.6	22.2	1.0 ± 0.3	1.1 ± 0.3^*^	0.4	10	0.6410	0.0002	0.6410
OLS right (s)	15.1 ± 8.9	25.6 ± 8.1	1.3	69.5	17.7 ± 12.2	21.8 ± 10	0.4	23.1	0.3694	0.0467	0.8654
Illinois test (s)	36.5 ± 6.0	31.8 ± 5.0^*^	0.9	12.8	35.3 ± 5.0	31.3 ± 4.3^*^	0.9	11.3	0.6148	0.0001	0.7256

* Main effect of time (*Post-hoc*), *p* < 0.05; AT, Agility training; BMI, body mass index; CT, Cognitive training; ES, effect size; IHG, isometric handgrip strength; OLS, One-leg stand; TUG, timed up-and-go test; WS, walking speed; 5XSTS, five-times sit-to-stand test.

**Figure 5:**
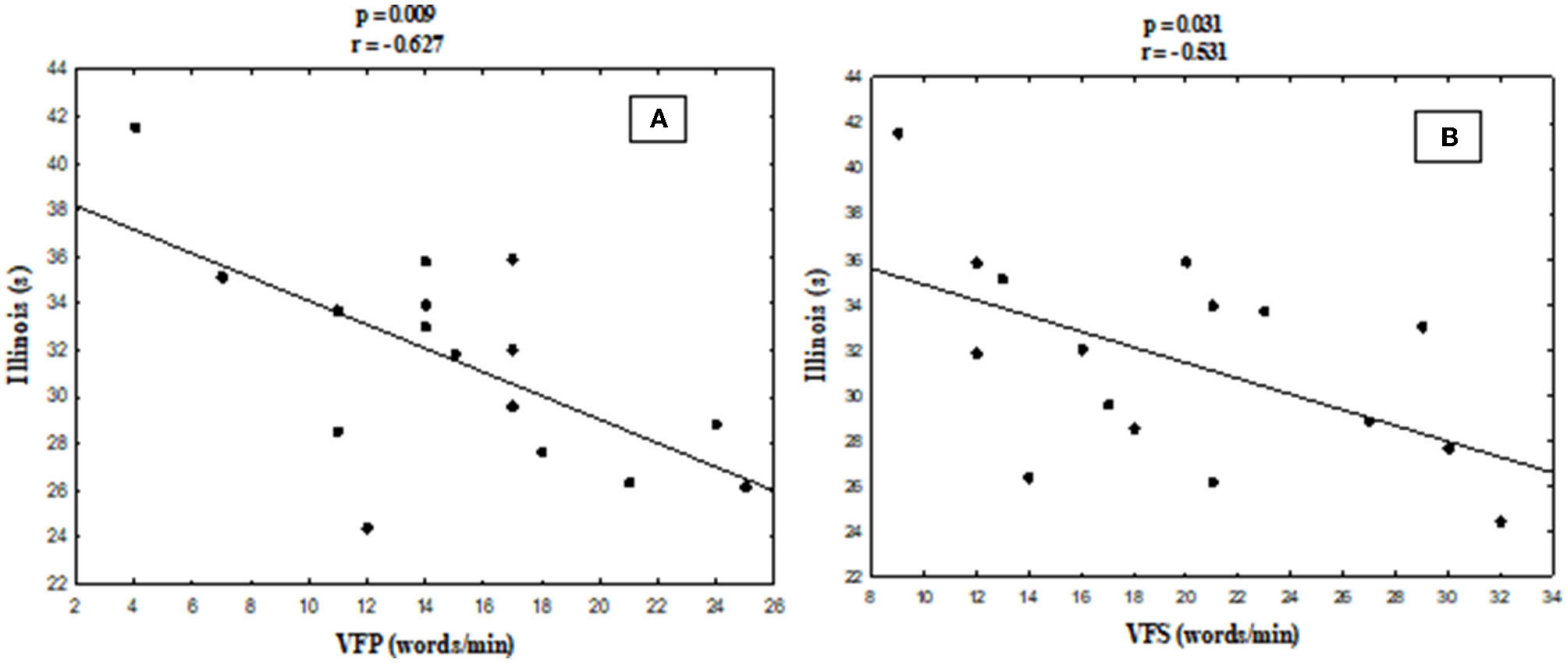
Pearson's correlation between the Illinois agility test and phonological **(A)** and semantic **(B)** verbal fluency scores.

There was a significant correlation between IAT scores and VFP (*r* = −0.627; *P* = 0.009, *n* = 16) and between IAT scores and VFS (*r* = −0.539; *P* = 0.031, *n* = 16) as seen in Figure 5.

## Discussion

The current study investigated the influence of 14-week AT and AT + CT on physical and cognitive functions in community-dwelling older adults. The current study shows that both interventions were beneficial for the physical function (in all the evaluated parameters) of the participants. Moreover, AT + CT had an additional positive effect on cognitive function (executive function and VF) in healthy older adults after 3 months of the intervention program (dual-task training). In addition, there was a positive correlation between agility test scores and VF scores, suggesting that those who performed better in the agility test (Illinois test) may have a high processing speed when evoking words.

### Cognitive functions

Cognitive functions showed improvements after the intervention program. TUG-cog scores were significantly higher in the AT + CT group. Previous studies support these results, wherein dual-task intervention (e.g., combined physical activity [PA] and CT) is more beneficial than PA or CT interventions alone (7, 13, 30, 31). Castaño et al. (6) showed that physical activity training for 16 weeks increased physical function. However, the authors showed that the combination of physical and cognitive training improved physical and cognitive function and increased the levels of neurotrophic biomarkers (brain-derived neurotrophic factor) (6). The cognitive functions improvement (e.g., short-term and working memory and executive function) may prevent the risk of cognitive impairment and dementia.

The participants who underwent AT + CT were required to perform VF (pronouncing specific classes of words) jointly with physical coordination tasks (agility training ladder), involving working memory and cognitive flexibility (32). A previous meta-analysis supports this finding and suggests that PA programs for older adults can yield superior cognitive benefits when cognitive tasks are integrated into the programs without interfering with the results of physical function (30).

Cognitive functions (including executive functions and memory) are essential in regulating functional mobility. Several studies have shown that gait abnormalities might precede the diagnosis of cognitive decline, and people showing slower gait during a dual task (e.g., TUG-cog) are more likely to develop cognitive impairment (27, 33). De Melo Borges et al. (27) showed that the greater the cognitive impairment, the worse the TUG-cog performance.

Physical activity can increase some cognitive functions, especially aerobic training, which has been shown to have the most significant impact on the aging brain and cognition compared to other types of physical activity (9, 13). SPMT scores increased in both groups, corroborating the findings in the literature. Although the intensity of aerobic training is essential for cognitive and physical improvements, aerobic training can counter cognitive declines, including memory (34).

### Physical function

The present results showed that agility ladder training with/without CT could prevent the loss of muscle power/strength despite the age-related decrease in neuromuscular performance (35, 36). Frailty is associated with decreased strength and physical function (37), and some studies demonstrated that physical activity can improve muscle power/strength and, finally, physical function in older adults (35–37).

The score in the TUG test is associated with the risk of falls in older adults (38). Segev et al. (39) found that a 12-week coordination training decreased the risk of falls in older adults with cardiovascular disease, demonstrating that a fast pace is related to a low risk of falls and better dynamic balance since those abilities are considered the main components of postural control.

The AT is comprised of perception (e.g., the organization, identification, and interpretation of sensory information), cognitive function (e.g., attention, planning, and decision-making), and changes in direction (e.g., sudden starts, stops, and turns, reactive control, and concentric and eccentric contractions) that might enable for integration of sensorimotor, neuromuscular, and cardio-circulatory demands (11, 40). Balance also depends on muscle strength and neuromuscular coordination (41), which may be one of the reasons why the interventions enhanced static and dynamic balance.

It is essential to highlight that AT + CT is low-cost, practical, readily accessible, and easily adapted to specific populations by changing speed and complexity (9). Studies attest to the beneficial effects of DT on executive function, fall prevention, and memory compared with isolated training (9, 42).

### Limitations

The limitations of this study include the lack of control and/or CT group and the small sample size, and the latter can potentially decrease statistical power. Despite not having a control group or one group that performed only CT, there was no significant difference between groups at the baseline, and we used their baseline data as control data. Although the sample size can be a limitation to be pointed out, especially regarding statistical power, the results found herein showed that the dual-task activity (physical and cognitive) was able to promote benefit in a greater proportion of the participants than the physical activity alone. It is a potential intervention that needs to be further explored.

## Conclusion

We investigated the effects of 14 weeks (two familiarization weeks) of AT and AT + CT on physical and cognitive function in community-dwelling older adults. Both interventions improved muscle power and strength, dynamic balance, agility, and short-term memory, whereas AT + CT showed additional improvements in other cognitive functions. Our results show that AT + CT should be included in physical activity programs for older adults because it is easy to apply, practical, and cost-effective intervention and has benefits for physical and cognitive function together.

## Data availability statement

The raw data supporting the conclusions of this article will be made available by the authors, without undue reservation.

## Ethics statement

The studies involving human participants were reviewed and approved by Institutional Human Research Ethics Committee of the UNICAMP. The patients/participants provided their written informed consent to participate in this study.

## Author contributions

VC and MU: conceptualization and project administration. LC and VC: data collection. LC, VC, and MU: formal analysis. VC, PS, RS, and MU: funding acquisition and methodology. LC, VC, and RS: investigation. CT and MU: supervision. LC, MU, and CT: writing—original draft. LC, CT, MU, PS, and RS: writing—reviewing and editing. All authors contributed to the article and approved the submitted version.
